# Diet Quality, Cardiometabolic Risk and Diabetes

**DOI:** 10.3390/nu15194283

**Published:** 2023-10-08

**Authors:** Giuseppe Della Pepa

**Affiliations:** 1 Department of Clinical Medicine and Surgery, Federico II University, Via Sergio Pansini 5, 80131 Naples, Italy; giuseppe.dellapepa@unina.it; 2 Cardiometabolic Risk Unit, Institute of Clinical Physiology, National Research Council-CNR, Via Giuseppe Moruzzi 1, 56124 Pisa, Italy

The alarming rise in obesity worldwide is a dramatic public health concern [1], and changes in lifestyle factors, such as low physical activity and the progressive diffusion of the high-calorie Western diet, are the major contributors to this trend [2]. The excess of fat accumulation, particularly abdominal fat, characterizing individuals with obesity drives the progression of multiple cardiometabolic risk factors and type 2 diabetes (T2D), which are the largest contributors to the global disease burden and disease-related mortality [2]. 

Weight loss achieved by different approaches, i.e., hypocaloric diet, pharmacotherapy, and bariatric surgery, represents the main therapeutic goal to treat obesity and prevent cardiometabolic risk factors and T2D [3]. However, these strategies might be unfeasible on a large scale and limited by some adverse effects [3]; furthermore, the long-term maintenance of weight reduction following restricted calorie diets represents a major challenge [4]. With this regard, body weight loss achieved by a hypocaloric diet tends to have rapid results in the early phase, followed by a plateau and a progressive weight regain in most individuals, attenuating the possible beneficial effect on cardiometabolic risk factors [5,6].

Changes in diet composition acting on nutrient quality independently of changes in energy intake may be effective in cardiometabolic and T2D risk prevention, offering a more feasible alternative treatment to energy restriction. Notably, this approach highlights the metabolic role of dietary components beyond their fuel value and introduces the concept of non-weight-loss-centered treatment of cardiometabolic risk factors associated with obesity [7,8].

Greater adherence to dietary patterns and/or consumption of dietary components linked to a preventive effect on cardiometabolic risk factors, T2D, or other chronic diseases, are two characteristics of higher diet quality [9]. The aim of the present Special Issue of *Nutrients*, entitled “Diet Quality, Cardiometabolic Risk, and Diabetes”, is to summarize recent evidence on the impact of diet quality in terms of dietary components (micro- or macronutrients) or dietary patterns on the prevention of cardiometabolic risk factors and T2D, beyond the effect of a restricted calorie diet and regardless of body weight loss.

Current evidence suggests that high-quality dietary patterns such as the Dietary Approaches to Stop Hypertension (DASH) eating plan and the Mediterranean diet are the most promising interventions to prevent cardiometabolic risk factors and T2D [10]. With this regard, Theodoris et al. [11], in their systemic review and meta-analysis performed on 12 epidemiological studies including more than 115.000 participants, showed that high adherence to the DASH diet reduced by ~20% the risk of hypertension compared with low adherence. These data strengthen and are in line with all hypertension guidelines, indicating that lifestyle changes focusing on diet quality should start early, even in populations with normal blood pressure. Interestingly, the DASH dietary pattern may be a promising target for T2D prevention efforts in young adults, considering that cardiometabolic risk factors and T2D are also dramatically increasing in this population [1]. In this way, Castello et al. [12] investigated the adherence to four dietary patterns and the risk for T2D in a cohort of primarily Hispanic young adults. The four dietary patterns were the DASH diet, the Healthy Eating Index (HEI), the Mediterranean diet, and the Diet Inflammatory Index (DII). The authors found that each one-point increase in DASH or HEI scores reduced the risk of prediabetes at follow-up by 64% and 9%, respectively [12].

In a very innovative way, Wang et al. [13] combined structural equation modeling (SEqM) and multivariate logistic regression to examine the association between dietary patterns and T2D in a large population of Asian individuals. The authors identified two dietary patterns: the modern dietary pattern (rich in red meats, vegetables, seafood, fungi and algae, main grains, and poultry, but low in whole grains and tubers) and the fruit-milk dietary pattern (rich in milk and its products, fruits, eggs, nuts and seeds, and pastry snacks but low in vegetable oils). Multivariate logistic regression and SEqM analysis revealed that only the modern dietary pattern was significantly associated with T2D [13].

Non-alcoholic fatty liver disease (NAFLD) and non-alcoholic steatohepatitis (NASH) are highly prevalent in T2D and are strongly associated with cardiometabolic risk factors [14]. Vitale et al. investigated in a large population of patients with T2D the association of different dietary components and food groups with NASH, in a real-life setting [15]. Authors reported that, despite the composition of macronutrients being similar in patients with or without NASH, diet quality was very different between groups; in fact, the intake of fiber, vitamins, and polyphenols was significantly lower in the NASH group. In terms of food groups, the NASH group was characterized by a lower consumption of whole grain bread, legumes and nuts, vegetables, fruits, fatty fish, milk and yogurt, and a higher consumption of pasta, rice, potatoes, red meat and processed meat [15]. These data indicate that overall, the dietary pattern in patients without NASH is close to the Mediterranean dietary model, contributing to the growing evidence suggesting this model as the reference nutritional pattern to prevent and treat NAFLD in people with T2D [16]. 

The health effects on cardiometabolic risk factors and T2D promoted by greater adherence to high-quality dietary patterns might be related to different dietary components such as fiber, n-3 and n-6 polyunsaturated fatty acids (PUFA), polyphenols, vitamins, and minerals. Among minerals, the exact role of selenium in glucose and lipid metabolism in patients with cardiometabolic disease remains undetermined, and in this Special Issue, Ouyang et al. presented data on this topic, performing a meta-analysis on 10 randomized controlled trials [17]. The findings reported that selenium supplementation significantly reduced values of fasting insulin and HOMA-IR, and increased high-density lipoprotein cholesterol, while it did not affect levels of fasting glucose, total cholesterol, triglycerides, low-density lipoprotein cholesterol, and very low-density lipoprotein cholesterol [17].

With regard to polyphenols, Popiolek-Kalisz investigated the impact of the long-term dietary intake of the selected flavonols and their main dietary sources on systolic and diastolic blood pressure in patients with cardiovascular disease (CVD). The authors reported that long-term dietary consumption of the flavonol isorhamnetin positively impacted on blood pressure in male patients, and the analysis of specific foods showed that onion, tomato, and blueberry were associated with this effect [18]. 

High-quality dietary patterns are also rich in phytosterols, and Witksowska et al. showed that the intake of total and individual plant sterols was lower among individuals with CVD compared with those without CVD. Furthermore, diet quality, as measured by the Healthy Diet Index, was significantly higher in the higher tertile of plant sterol intake [19].

Greater adherence to high-quality dietary patterns favorable impact cardiometabolic risk factors and T2D by different mechanisms, and the potential ability to decrease inflammation and oxidative stress emerged as one of the leading candidate hypotheses [20]. The DII was a dietary assessment tool developed to estimate the inflammatory potential of a diet [21]. A high DII score, which was associated with elevated inflammatory markers, indicates a pro-inflammatory diet and has been reported to be correlated with cardiometabolic risk factors and T2D [22]. In a very large American population, Yuan et al. investigated the long-term prognostic value of DII, showing that individuals with prediabetes and T2D had higher DII scores compared with individuals with normoglycemia [23]; furthermore, individuals with high DII scores were at a higher risk of long-term all-cause mortality (HR: 1.59, 95% CI: 1.37, 1.86) and cardiovascular mortality (HR: 2.04, 95% CI: 1.45, 2.84) compared with those with a lower DII [23]. These data call for a comprehensive assessment of the dietary inflammatory potential in patients with T2D.

With regard to oxidative stress, the oxidative balance score (OBS) evaluates the oxidative balance of the lifestyle pattern of an individual in terms of the incorporated consumption of anti- and pro-oxidants [24]; among dietary components, the OBS considers lower intakes of saturated fatty acids, n-3 PUFA, iron, and higher intakes of vitamin C, vitamin E, selenium, and β-carotene [24]. In their longitudinal study, Kwon et al. reported that the hazard ratio for T2D incidence of the highest tertile groups of OBS, compared with the referent lowest tertile group, was 0.83 in men and 0.78 in women, respectively [25]. This current study is significant for providing evidence of a positive relationship between OBS score and T2D incidence risk, highlighting the importance of an antioxidant-rich diet.

Finally, the last article of this Special Issue emphasizes the importance of remote interventions, as we have learned from the last pandemic. Remote interventions can reach different people, get around obstacles and hurdles, and offer strategies for promoting greater adherence to high-quality dietary patterns and/or components. In this way, in their randomized controlled trial, Khol et al. evaluated the effects of two different web-based weight loss programs on diet quality as assessed by the HEI [26]. The intervention group received a fully automated and interactive web-based program focusing on dietary energy density, while the control group was exposed to a non-interactive web-based program that addressed the same topics. The authors show that the interactive and fully automated web-based weight loss program improved the diet quality of the participants [26]. These data show the importance of remote intervention in improving diet quality for people who cannot have access to personal care.

In summary, focusing on a few modest dietary changes in terms of diet quality, such as increasing the consumption of whole grain products, legumes and nuts, vegetables, fruits, fatty fish, milk and yogurt, and extra virgin olive oil, and reducing the consumption of refined cereal products, red meat and processed meat, might have a beneficial impact on cardiometabolic health. These effects can be achieved regardless of body weight loss and avoiding the too-radical strategies of restrictive hypocaloric dietary approaches, which might be limited by long-term adherence and body weight loss regain. 

In this regard, the DASH eating plan and the Mediterranean diet are proven examples of successful strategies for the prevention of cardiometabolic risk factors and T2D.

Further studies are needed to fully elucidate the mechanisms by which high-quality dietary patterns and dietary components might act on the prevention of cardiometabolic risk factors and T2D. A better understanding of these pathways may be essential in order to promote a personalized dietary approach for the cardiometabolic health of patients with obesity.
